# Loss of PTEN-assisted G2/M checkpoint impedes homologous recombination repair and enhances radio-curability and PARP inhibitor treatment response in prostate cancer

**DOI:** 10.1038/s41598-018-22289-7

**Published:** 2018-03-02

**Authors:** W. Y. Mansour, P. Tennstedt, J. Volquardsen, C. Oing, M. Kluth, C. Hube-Magg, K. Borgmann, R. Simon, C. Petersen, E. Dikomey, K. Rothkamm

**Affiliations:** 1 Laboratory of Radiobiology and Experimental Radiooncology, University Medical Center Hamburg-Eppendorf, Hamburg, Germany; 2 Department of Tumor Biology, National Cancer Center, Cairo, Egypt; 3 Martini-Clinic, University Medical Hamburg Eppendorf, Hamburg, Germany; 4 Department of Oncology, Hematology and Bone Marrow Transplantation, University Medical Center Hamburg-Eppendorf, Hamburg, Germany; 5 Department of Pathology, University Medical Center Hamburg-Eppendorf, Hamburg, Germany; 6 Department of Radiotherapy and Radiooncology, University Medical Center Hamburg-Eppendorf, Hamburg, Germany

## Abstract

Here we report that PTEN contributes to DNA double-strand break (DSB) repair via homologous recombination (HR), as evidenced by (i) inhibition of HR in a reporter plasmid assay, (ii) enhanced sensitivity to mitomycin-C or olaparib and (iii) reduced RAD51 loading at IR-induced DSBs upon PTEN knockdown. No association was observed between PTEN-status and RAD51 expression either *in-vitro* or *in-vivo* in a tissue microarray of 1500 PTEN-deficient prostate cancer (PC) samples. PTEN depletion and sustained activation of AKT sequestered CHK1 in the cytoplasm, thus impairing the G2/M-checkpoint after irradiation. Consistently, AKT inhibition recovered the G2/M-checkpoint and restored HR efficiency in PTEN-depleted cells. We show that, although *PTEN* loss correlates with a worse prognosis, it may predict for improved response of PC patients to radiotherapy. Further, we provide evidence for the use of PTEN as a biomarker for predicting the response to PARP inhibitors as radiosensitizing agents in prostate cancer. Collectively, these data implicate PTEN in maintaining genomic stability by delaying G2/M-phase progression of damaged cells, thus allowing time for DSB repair by HR. Furthermore, we identify PTEN-status in PC as a putative predictor of (i) radiotherapy response and (ii) response to treatment with PARP inhibitor alone or combined with radiotherapy.

## Introduction

Among the different types of DNA damage, DNA double-strand breaks (DSBs) are the most common cause of genomic instability and tumor formation and are also the decisive product of various anti-tumor therapies including radio- and most chemotherapies. Mammalian cells repair DSBs by two major repair pathways referred to as non-homologous end joining (NHEJ) and homologous recombination (HR)^1^. NHEJ is a fast process and represents the major DSB repair pathway in mammalian cells, repairing DSBs in all cell cycle phases but predominantly in G1^2^. HR is a comparatively slow repair process which is restricted to S/G2 phase when the intact sister chromatid is available as a template to allow error-free repair. We have previously described a functional hierarchy in DSB repair^3^, which regulates the choice between DSB repair pathways. Deregulation of this hierarchy has been described in several tumors^4–6^, leading to deficient repair and genetic aberrations. Generally, deregulation of this hierarchy might be caused by altered expression of certain oncogenes or loss of tumor suppressor genes (TSGs). Such a disturbance in the balance between the different DSB repair pathways in cancer has been used to specifically target tumor cells, avoiding normal tissue toxicity, i.e. through synthetic lethality^7^. We and others have previously shown that mutations in specific TSGs are synthetic lethal with inhibition of the DNA repair enzyme poly(ADP-ribose) polymerase 1 (PARP1)^5,8–14^.

PTEN (Phosphatase and tensin homolog deleted on chromosome 10) was identified as a TSG on chromosome 10q23. PTEN encodes a dual-specificity phosphatase that functions as (i) a direct antagonist of phosphatidylinositol 3-kinase and (ii) a key kinase involved in AKT activation^15^. *PTEN* alterations have been implicated in various human cancers including prostate cancer^16,17^. Inactivation of PTEN causes constitutively activated levels of AKT, thus promoting cell growth, proliferation, survival, and migration through multiple downstream effectors. However, additional evidence suggested other functions for PTEN that are unrelated to PI3-K/AKT signaling, specifically in DNA damage repair. Involvement of PTEN in the repair of DSB has been the subject of a few studies in the last decade. PTEN deletion in mouse embryonic fibroblasts (MEFs) causes spontaneous DSBs and genomic instability^18^. Shen and coworkers attributed this role to the ability of PTEN to regulate the expression of RAD51, the main player of HR repair. Several other reports indicated that reduced levels or deletion of PTEN are associated with decreased HR efficiency^18–21^. However, these data could not be confirmed using a different cell type^16^ or even using the same cell system^22^, arguing against a direct role for PTEN in DSB repair. Thus the precise role of PTEN in DSB repair needs to be further characterized in more detail.

In the current study, we report that PTEN contributes to DSB repair by HR through regulating the CHK1-mediated checkpoint to give cells time to repair the induced DSBs before entering mitosis. This data defines a link between PTEN and HR function especially in prostate cancer. Interestingly, here we report that PTEN deletion might be used as a predictor for the response to radiotherapy in prostate cancer (PC), which may help inform treatment decisions. Furthermore, the suggested role of PTEN in HR would support the treatment of PTEN-deficient PC patients with agents targeted against defects in HR such as PARP inhibitors (PARPi).

## Results

### PTEN depletion diminishes DSB repair via homologous recombination

**Figure 1:**
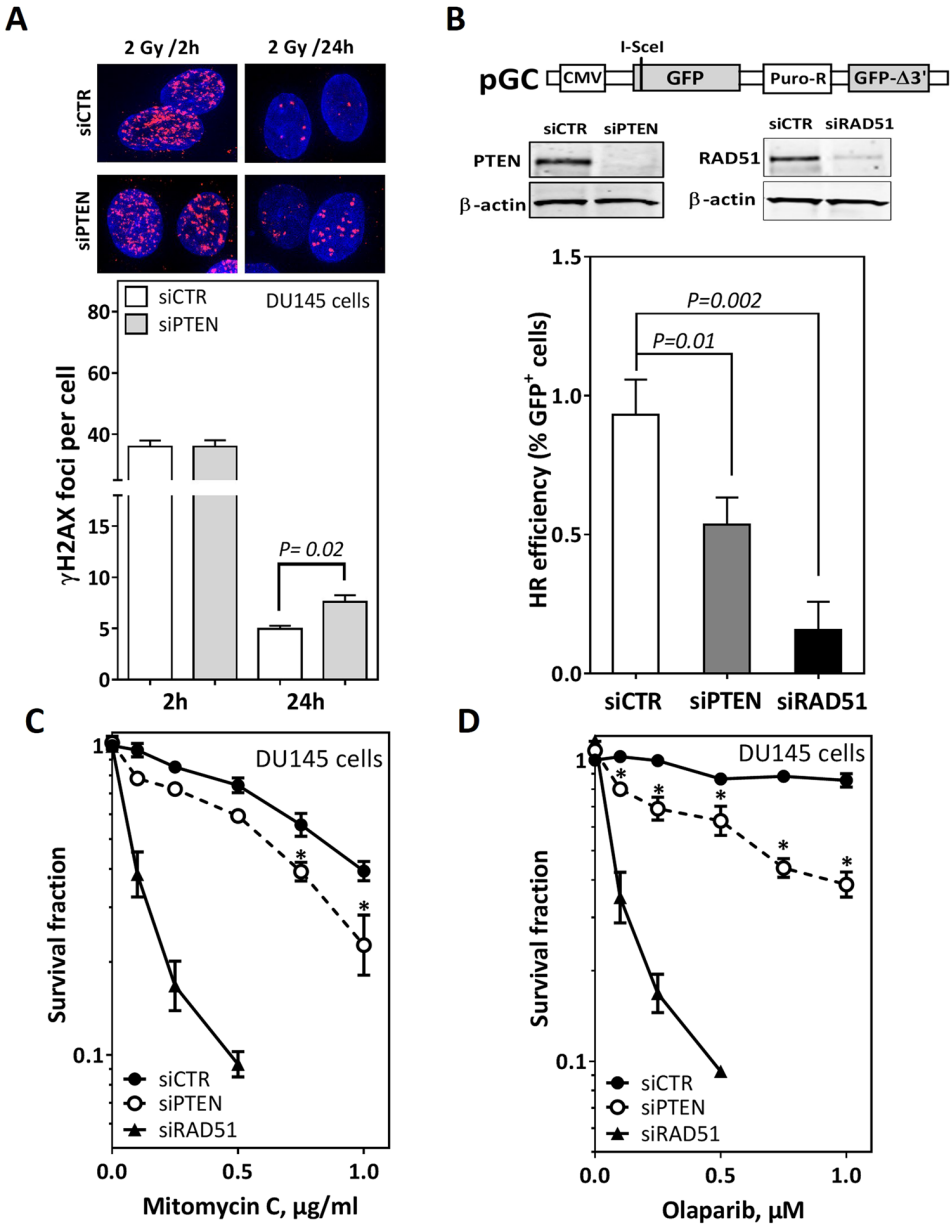
PTEN depletion diminishes DSB repair via homologous recombination. (**A**) yH2AX foci (red) at the indicated times after 2 Gy in mock- (siCTR) or PTEN-depleted DU145 cell nuclei (blue). (**B**) PTEN was efficiently depleted (insert) in HeLa cells harboring single copies of the HR-substrate pGC. After 24 h, cells were transfected with I-SceI expressing vector to induce DSB and the percentage of GFP-positive (GFP^+^) cells was measured using flow cytometry as an indicator of HR efficiency. (**C**–**D**) Survival fractions after treatment with the indicated concentrations of mitomycin C (**C**) or the PARP inhibitor olaparib (**D**) were measured in mock- (siCTR) or PTEN- depleted DU145 cells (siPTEN) using the colony forming assay. RAD51 was depleted using siRNA (siRAD51) as a positive control in all experiments. Shown are means ± SEM of three independent experiments. Asterisk (*) represents significant difference (P < 0.05).

We sought to investigate whether PTEN is involved in the repair of DSBs. To that end, we depleted PTEN in DU145 cells before irradiation with 2 Gy. γH2AX foci were monitored at 2 h and 24 h after irradiation as a readout for DSB induction and repair, respectively (Fig. 1A, upper panel). While the number of γH2AX foci is similar at 2 h, it is 50% higher in PTEN-depleted cells (7.6 ± 0.66 foci/cell) after 24 h compared to their wildtype counterparts (4.9 ± 0.31 foci/cell) (Fig. 1A, lower panel), indicating a role for PTEN in DSB repair. This role was not associated with impaired ataxia-telangiectasia mutated (ATM)-signaling as evidenced by normal recruitment of pATM and its downstream target BRCA1 in both PTEN-depleted and wildtype cells (Supplementary Figure S1A,B). Next, we sought to directly measure HR using a reporter assay in PTEN-depleted cells. To that end, using siRNA PTEN was efficiently depleted in HeLa cells harboring single copies of pGC (HeLa-pGC) (Fig. 1B), a previously validated HR substrate^3^. Depletion of PTEN resulted in about 30% reduction in DSB repair via HR when compared to the wild type cells (Fig. 1B). Expectedly, depletion of the main HR player RAD51 dramatically reduced HR efficiency (Fig. 1B). An important prediction from the observed HR deficiency is that PTEN-depleted cells should be sensitive to drugs that generate replication- associated DSBs such as mitomycin-C (MMC) and the PARP inhibitor olaparib^5,12^. Consistently, PTEN-depleted DU145 cells were significantly more sensitive to MMC (Fig. 1C) or olaparib (Fig. 1D) than their wild type counterparts, indicating an important role for PTEN in HR. This has been also confirmed in the benign prostate cell line BPH1 (Supplementary Figure S1C). Again, RAD51 depletion caused severe sensitivity to MMC and olaparib in both cell lines (Fig. 1C/D and Supplementary Figure S1C). Notably, depletion of PTEN did not alter NHEJ efficiency measured in HeLa cells harboring the NHEJ-substrate pEJ (Supplementary Figure S2). Together, these data indicate that PTEN is involved in the repair of DSB via HR and that PARPi treatment should be considered for patients with PTEN-deleted PC.

### PTEN contributes to efficient RAD51 loading after ionizing radiation

**Figure 2:**
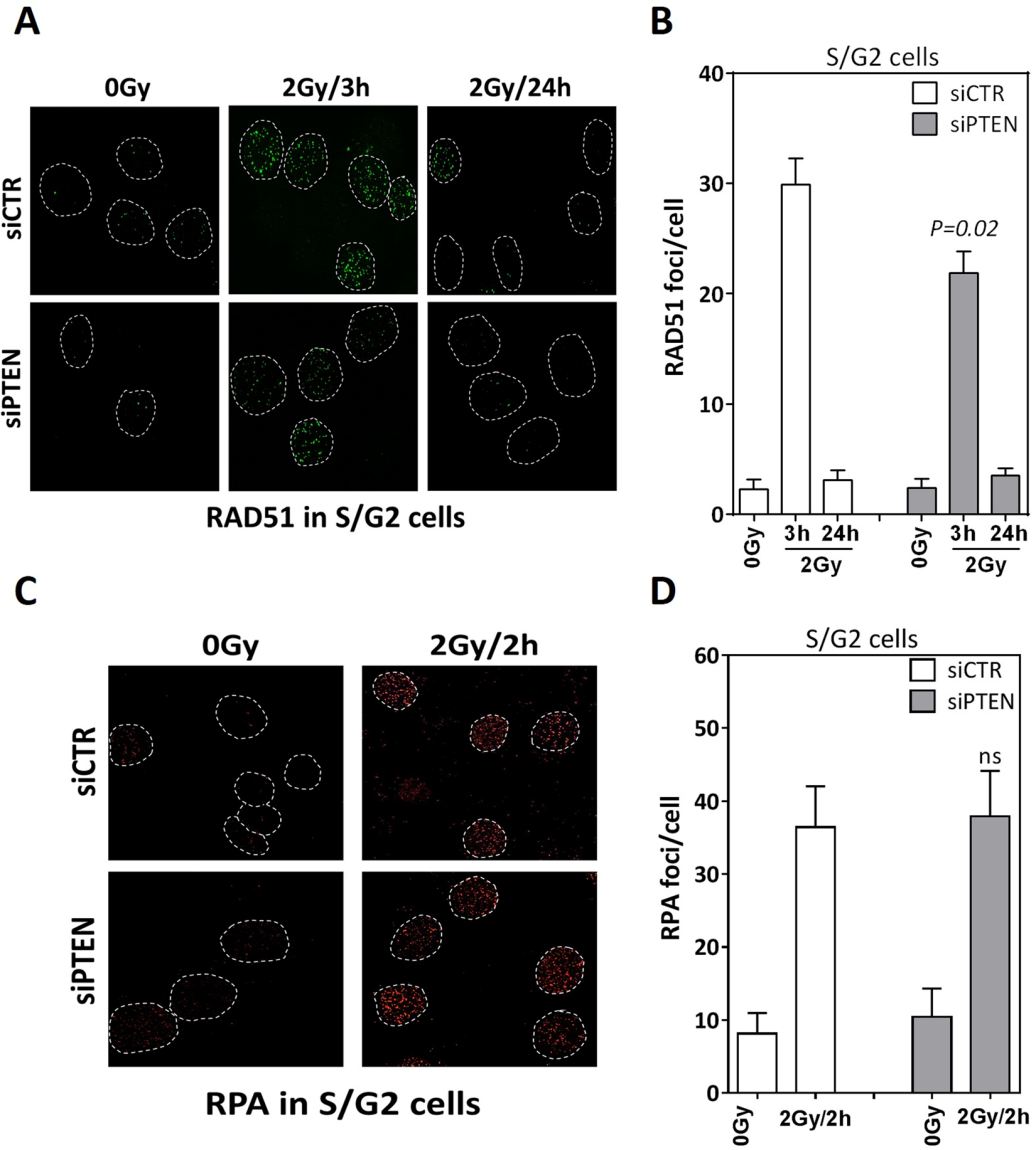
PTEN depletion decreases RAD51 loading. (**A**) RAD51 foci (green) at 3 h and 24 h after 2 Gy in CENPF-positive S/G2-phase Du145 cells transfected by either scrambled siRNA (siCTR) or siRNA targeting PTEN (siPTEN). (**B**) Quantitation of A. (**C**) RPA foci (red) at 2 h after 2 Gy in CENPF-positive S/G2-phase DU145 cells transfected by either scrambled siRNA (siCTR) or siRNA targeting PTEN (siPTEN). (**D**) Quantitation of the experiments performed in C. For IF experiments, at least 50 cells were analyzed. Shown are the means ± SEM from at least three different experiments. ns: not statistically significant.

Mendes-Pereira and colleagues previously showed that PTEN loss reduced the formation of DSB-induced RAD51 foci^20^; however, other studies showed that RAD51 foci are induced normally in cells lacking PTEN^19,22^. To assess whether or not PTEN affects the recruitment and/or clearance of RAD51 at DSB, we depleted PTEN in DU145 cells and treated them with 2 Gy before evaluating RAD51 foci at IR-induced DSBs at 3 h (as an indication for the loading efficiency) and 24 h (as a read-out for completion of the repair by HR) by immunofluorescence (Fig. 2A). As shown in Fig. 2B, wild type cells have efficiently loaded RAD51 3 h post irradiation, with a subsequent decline to the normal level after 24 h, indicating efficient HR repair. However, in PTEN-depleted cells the loading of RAD51 was modestly but significantly (P = 0.02) reduced compared to wild type cells, but interestingly all RAD51 foci were cleared after 24 h. This data indicates that PTEN may play a role in loading of RAD51 at the DSBs. In order to unravel this role, we sought to analyze whether PTEN is involved in DSB end resection which is a pre-requisite step for RAD51 loading. To that end, we irradiated DU145 cells after PTEN depletion and monitored RPA (replication protein A) foci formation at 2 h after 2 Gy as a surrogate for end resection (Fig. 2C). Interestingly, downregulation of PTEN did not affect the formation of RPA foci in response to IR (Fig. 2D), indicating that the reduction in RAD51 loading in PTEN-deficient cells is not due to impaired DSB end resection.

**Figure 3:**
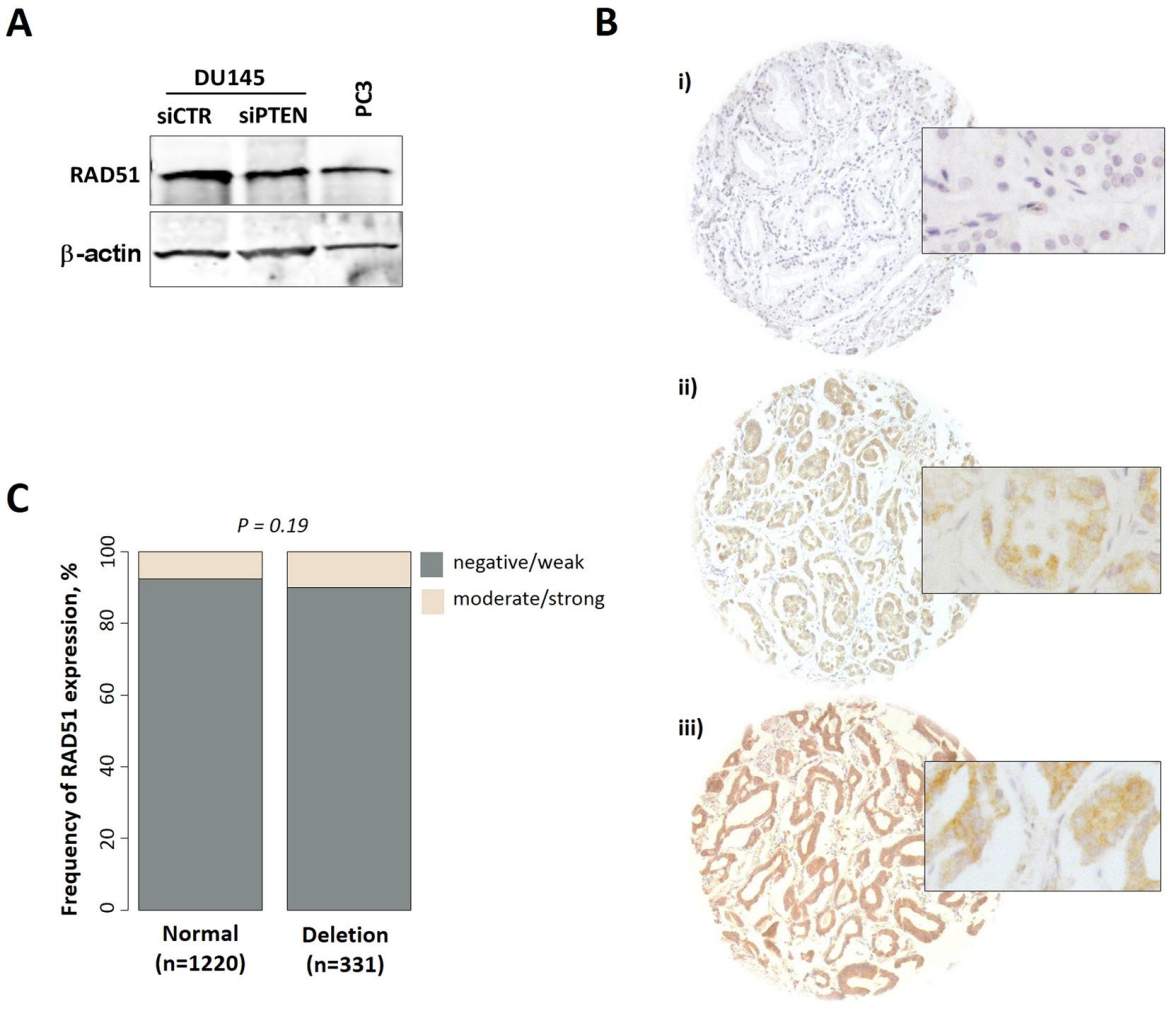
No correlation between PTEN status and RAD51 expression. (**A**) Western blot showing RAD51 expression in PTEN-proficient (siCTR) and -depleted (siPTEN) DU145 and PTEN-knockout PC3 cells. (**B**) Representative images of RAD51 immunostaining showing (i) negative/weak, (ii) moderate and (iii) strong staining. (**C**) Frequency of RAD51 expression level in PTEN-normal (n = 1220) or PTEN-deleted (n = 331) PC patients.

Several studies have shown that PTEN is important for maintaining basal levels of transcription of the RAD51 gene^18,20^, providing a potential mechanism to explain the impairment of RAD51 loading at DSBs and the reduced HR efficiency (Fig. 1B). We tested this possibility by analyzing the expression level of RAD51 in PTEN-depleted DU145 or PTEN-knockout PC3 cells. Interestingly, no significant changes in RAD51 protein levels were observed in PTEN-deficient compared to wildtype cells (Fig. 3A). For further confirmation, we investigated RAD51 expression using IHC (Fig. 3B) in a tissue microarray of PC with normal (n = 1220) or deleted PTEN (n = 331). IHC analysis of RAD51 expression showed no correlation between PTEN status and RAD51 expression (Fig. 3C), suggesting that PTEN regulates HR through a different mechanism.

### Defective CHK1-mediated G2-M checkpoint in PTEN-deficient cells

**Figure 4:**
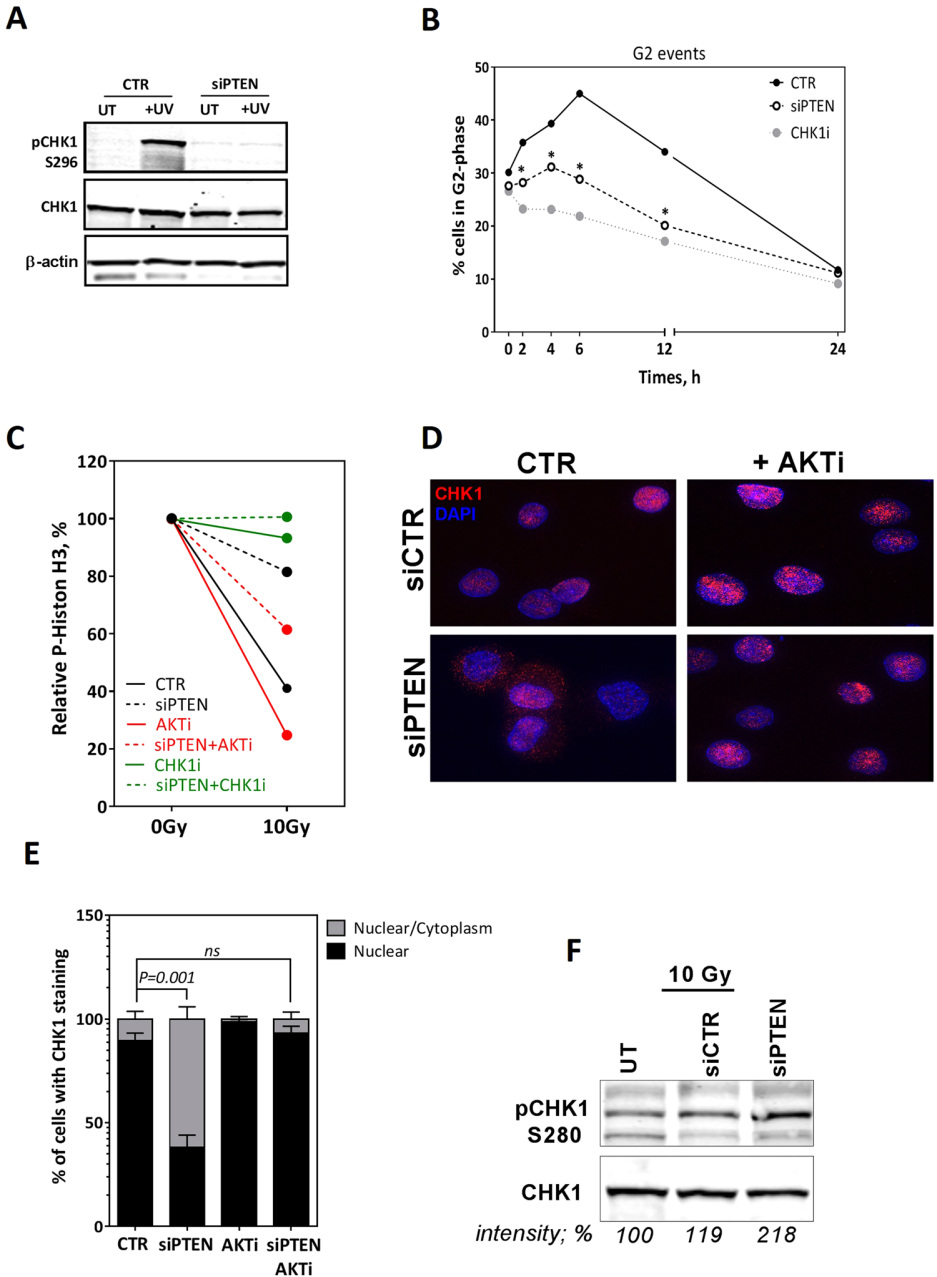
PTEN contributes to the G2/M checkpoint. (**A**) DU145 cells were transfected with either scrambled (CTR) or PTEN (siPTEN) siRNA. After 48 h, cells were UV-irradiated (50 J/m^2^) and the CHK1 auto-phosphorylation site (S296) was immunoblotted. Both ß-actin and CHK1 were used as loading controls. (**B**) Percent of DU145 cells in G2-phase was monitored using propidium iodide (PI) staining by flow cytometry after treatment with scRNA, siPTEN or the CHK1 inhibitor UCN01 (50 nM). Asterisk (*) represents significant difference (P < 0.05). (**C**) After the indicated treatments, DU145 cells were irradiated with 10 Gy, treated with colcemid to accumulate mitotic cells and then co-stained with phosphohistone H3 (S10) antibody and PI to detect mitotic cells. Shown are the means ± SEM from three experiments. (**D**) Immunofluorescence microscopy of CHK1 2 h after 2 Gy in siRNA-treated DU145 cells. (**E**) Quantitation of subcellular localization of CHK1 presented in D. Shown are the means ± SEM from at least three different experiments. ns: not statistically significant. (**F**) Western blot showing pCHK1-S280 and CHK1 in DU145 transfected with siCTR or siPTEN. Band intensities (measured by UNSCAN-IT gel V6.1) in percentage are shown.

Our data support the idea that PTEN is involved in the recruitment of RAD51 to the DSBs after IR and that is why HR is impaired in PTEN-depleted cells. Previously, it was shown that lack of PTEN impairs the CHK1-mediated G2-M checkpoint, initiating genetic instability^22^, possibly by not allowing cells enough time to execute HR following DNA damage. In order to address this possibility, we examined the CHK1 activity in PTEN-depleted cells. Briefly, DU145 cells depleted for PTEN were UV-irradiated with 50 J/m^2^ and phosphorylation of CHK1 at S296 (auto-phosphorylation site) was monitored using immunoblotting. As shown in Fig. 4A, although wild type cells responded well to UV irradiation by increasing the signal of pCHK1 (S296), the signal is profoundly diminished in PTEN-depleted cells. Indeed, impaired CHK1 phosphorylation was recapitulated after irradiating cells with 10 Gy X-rays (Supplementary Figure S3A), indicating that CHK1-signaling is compromised in PTEN-deficient cells. Given CHK1’s role in the G2/M checkpoint, we next examined the ability of PTEN-deficient cells to respond to IR and accumulate in G2 phase. To address this, DU145 cells depleted for PTEN were irradiated with 5 Gy and G2-phase cells were measured using flow cytometry. We consistently observed that PTEN-depleted cells did not arrest in G2 as efficiently as wildtype cells (Fig. 4B). About 45% of wildtype cells accumulated in G2 phase at 6 h after irradiation whereas only 27% of cells did so after PTEN depletion. Essentially, CHK1 inhibition showed a similar effect as PTEN-depletion on G2 accumulation. In order to further confirm our findings, PTEN-depleted DU145 or PTEN-knockout PC3 cells were irradiated with 10 Gy and treated with colcemid to collect cells in mitosis. After 2 h, cells were stained for the mitotic marker phospho-histone H3 (pH3) as a read-out for mitotic index. While wildtype cells showed an efficient decrease in pH3 signal after irradiation, PTEN-depleted cells exhibited inefficient reduction in pH3 signal (Fig. 4C), indicating an impaired G2/M checkpoint in PTEN-depleted cells. As expected, CHK1 inhibition also diminished G2/M checkpoint function (Fig. 4C and Supplementary Figure S3). Together, these data indicate that PTEN is required for CHK1 activity and hence for an optimal G2/M checkpoint after IR.

PTEN is a phosphatase that regulates AKT signaling^15^. We next addressed the question whether PTEN controls the G2/M checkpoint through regulating AKT activity. As expected, AKT phosphorylation (pAKT S473) was found to be upregulated in DU145 cells after PTEN depletion as well as in PTEN knockout PC3 cells (Supplementary Figure S4A). Interestingly, inhibition of AKT using CAS612847-09-3 (5 µM) in either PTEN-depleted DU145 cells or PTEN-knock out PC3 cells rescued the G2/M checkpoint as evidenced by reduction in pH3 signal (Fig. 4C and Supplementary Figure S3, respectively). This data suggests that PTEN regulates G2/M checkpoint through the AKT signaling pathway.

### PTEN is an important regulator of CHK1’s cellular localization

AKT phosphorylates CHK1 at S280, thus regulating CHK1’s cellular localization^22^. Upon DNA damage, CHK1 protein translocates into the nucleus, where it can be activated by ATM and ATR kinases to initiate the checkpoint response^23^. This might explain the impaired G2/M checkpoint in PTEN-depleted cells. In order to determine whether loss of PTEN and concomitant sustained activation of AKT alter the subcellular localization of CHK1, PTEN was depleted in DU145 cells before irradiation with 2 Gy and CHK1 subcellular localization was monitored by indirect immunofluorescence (Fig. 4D). After IR, CHK1 was almost exclusively detected in the nucleus in normal cells. However, in PTEN-depleted cells CHK1 showed a pan-cellular localization, being present in both nucleus and cytoplasm. While it did not alter the cellular distribution of CHK1 in wildtype cells, inhibition of AKT reverted CHK1′s cellular distribution in PTEN-depleted cells to normal (Fig. 4D,E). Consistent with this and with the findings of Puc *et al*.^24^, AKT-dependent phosphorylation of CHK1 (i.e. at S280) was found to be enhanced in PTEN-depleted cells (Fig. 4F). Together, these data suggest that PTEN controls the subcellular localization of CHK1 and hence the G2/M checkpoint through regulating the AKT activity.

### The defective G2/M checkpoint is responsible for the reported HR deficiency

**Figure 5:**
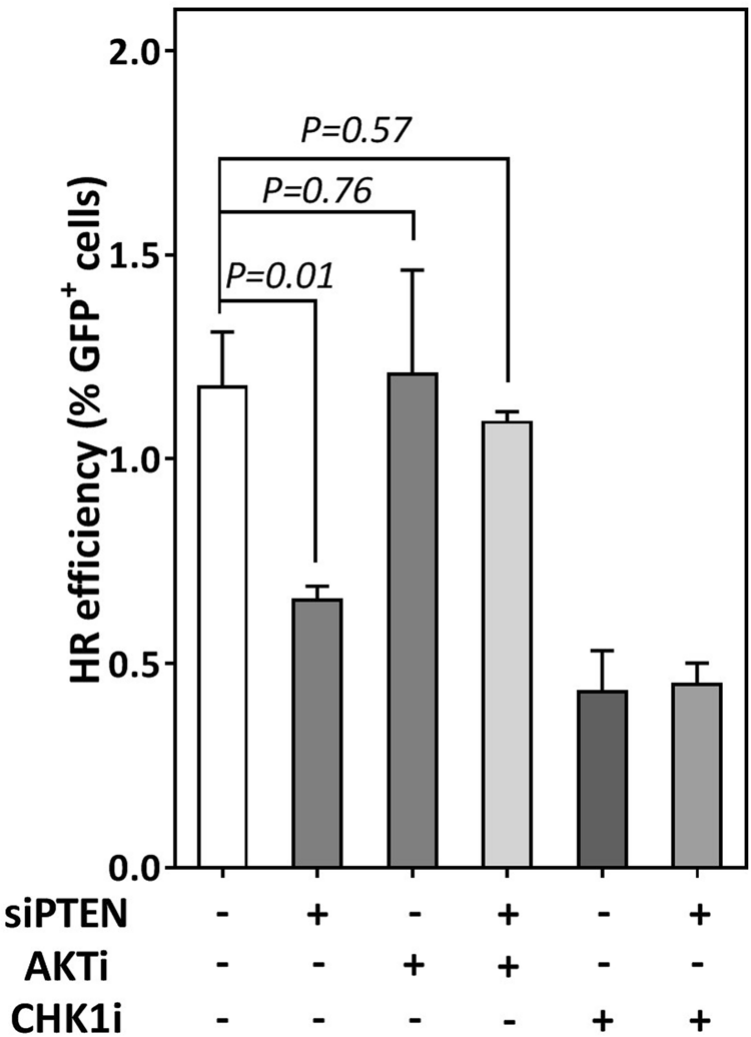
HR efficiency was rescued in PTEN-depleted cells after AKT inhibition. HR was measured, as described in Fig. 1, after the indicated treatments in HeLa cells harboring the HR substrate pGC. Shown are the means ± SEM from three experiments.

The above findings indicate that the PTEN-AKT pathway regulates the CHK1-mediated G2/M checkpoint which might explain the HR defect reported in PTEN-depleted cells. To address this, we sought firstly to analyze whether PTEN phosphatase activity is required for efficient HR. To that end, PTEN was inhibited in HeLa-pGC cells by treatment with either bpV(HOpic) (14 nM) or SF1670 (2 µM) and HR was measured as described above. As illustrated in Supplementary Figure S4B, inhibition of PTEN resulted in suppression of HR comparable to PTEN knockdown. Consistently, MMC sensitivity was enhanced in DU145 cells treated with either inhibitor (Supplementary Figure S4C). We reasoned that if PTEN regulates HR via its AKT-dependent role in the G2/M checkpoint, then inhibition of AKT would rescue HR in PTEN-depleted cells. To verify this, PTEN was depleted in HeLa-pGC cells and HR efficiency was measured before and after AKT inhibition. Again, depletion of PTEN diminished HR efficiency, which confirms our data presented in Fig. 1. Interestingly, HR efficiency was significantly rescued in PTEN-depleted cells after inhibition of AKT (Fig. 5), suggesting that PTEN regulates HR efficiency likely through regulating the AKT-mediated G2/M checkpoint.

### PTEN depletion renders cells radiosensitive and susceptible to radiosensitization by PARP inhibition

**Figure 6:**
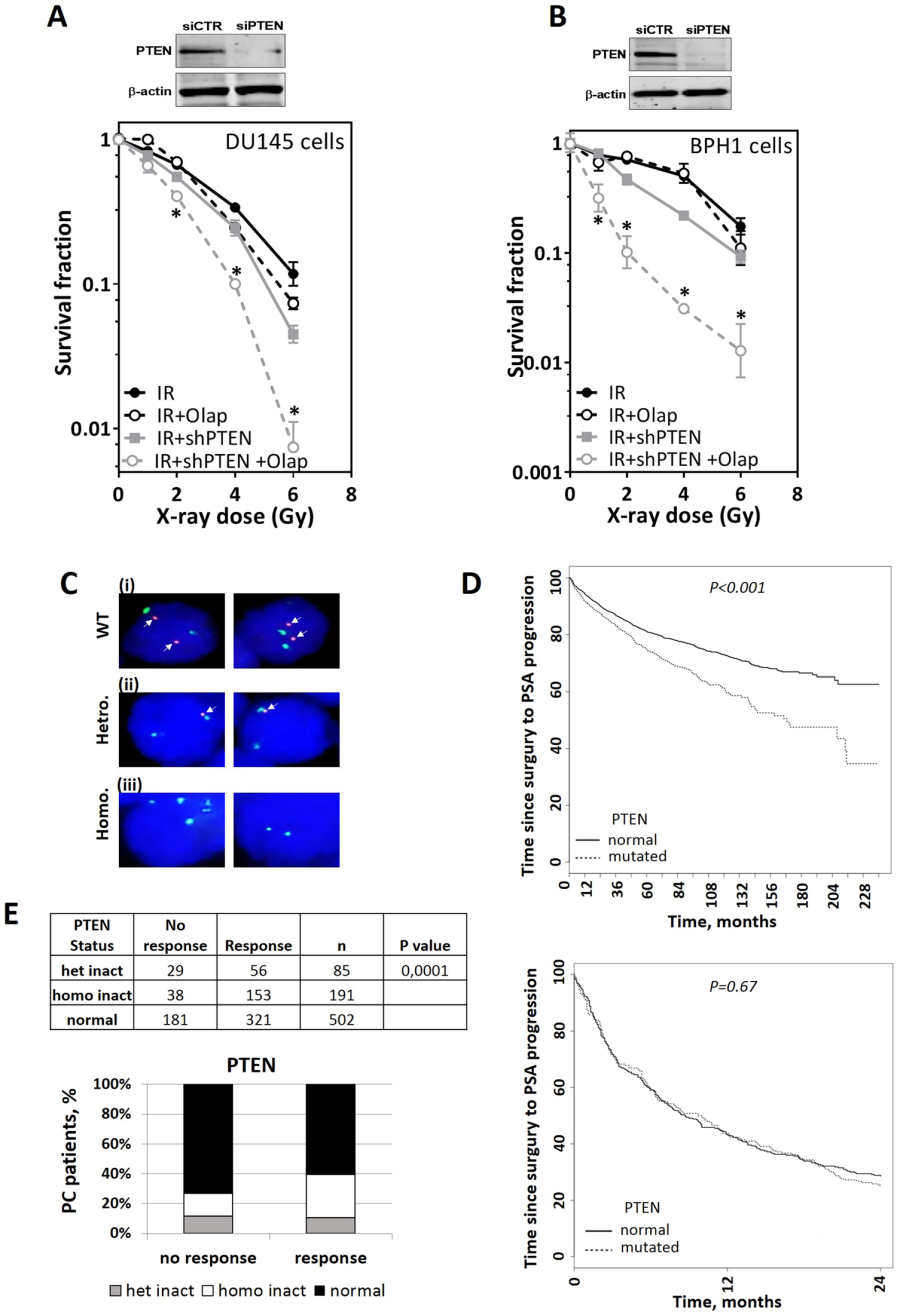
PTEN depletion renders cells susceptible to radiosensitization by PARP inhibition. (**A**–**B**) Stably PTEN-depleted DU145 or BPH1 cells (shPTEN) were treated with either DMSO or 1 µM of olaparib (Olap) prior to exposure to the indicated doses of ionizing radiation. The surviving fractions were then calculated by colony forming assay (see Materials and Methods). Shown are the means ± SEM from three experiments. Asterisk (*) represents significant difference (P < 0.05). (**C**) FISH was used to detect genomic PTEN deletions. Examples of PTEN status detected by FISH are shown (i-iii). (i) Wild type (WT) PTEN with normal copy number with two red *PTEN* signals and two green signals of centromere 10. (ii) Heterozygous deletion (hetro.) of *PTEN* showing loss of one red signal but two green centromere 10 signals. (iii) Homozygous deletion (homo.) of PTEN showing no red signal but two green signals. (**D**) Percentage of prostate cancer patients with or without PTEN deletion who show a decrease in PSA level (<0.2ng/ml) after radiotherapy. Correlation was measured by univariate analysis. (**E**) Multivariate analysis showing that PTEN status is not a predictive marker for radiotherapy response.

Our data support the hypothesis that PTEN status affects HR-mediated DSB repair. Thus, PTEN loss could be associated with increased sensitivity to irradiation. To address this issue, pMKO.1 puro PTEN shRNA (Addgene plasmid # 10669) was stably integrated in DU145 and BPH1 cells. PTEN depletion in DU145 cells significantly increased sensitivity to irradiation (Fig. 6A). More importantly, olaparib treatment further radiosensitized PTEN-depleted DU145 (Fig. 6A) and BPH1 cells (Fig. 6B). Together, these data suggest that tumors with loss or downregulation of PTEN may be amenable to treatment with PARP inhibitors in combination with irradiation.

The correlation between PTEN status and PC patient outcome was next investigated. To that end a 20-year follow-up study was conducted in a total cohort of 2,385 patients undergoing radical prostatectomy. Confirming previously published data, PTEN loss was identified as an independent prognostic marker of PSA recurrence-free survival in PC patients^25^. Given that PTEN depletion sensitizes cells to IR (Fig. 6A,B), we sought to analyze the possibility to use PTEN status as a predictive marker for the response to RT. To that end, PTEN status was examined using FISH (Fig. 6C) in 778 PC patients of the aforementioned group, who had been given RT due to rising PSA values following radical prostatectomy at the University Medical Center Hamburg-Eppendorf. Intriguingly, univariate analysis showed that PTEN deletions are significantly more frequent in patients who showed a better response after RT, defined as a decrease of PSA levels below 0.2 ng/ml. However, after adjustment by CAPRA-S^26^ and age (multivariate analysis) no difference was found for time to PSA recurrence (PSA ≥0.2 ng/ml) after radiation therapy between patients with identified PTEN mutation and normal PTEN status (Fig. 6E). Together, this indicates that although it may not be an independent predictive marker, PTEN loss can potentially be used to predict the response of PC patients to radiotherapy.

## Discussion

The role of PTEN in HR has been addressed in several studies; however, no concrete conclusion can be derived due to discrepancies between reports and in some cases even within the same study. While some reports revealed a critical, direct role for PTEN in the HR repair pathway^19–21^, other studies excluded completely any role for PTEN in HR^16,27^. Mendes-Pereira and colleagues showed that PTEN deficiency causes an HR defect in tumor cells which sensitizes them to PARPi both *in vitro* and *in vivo*^20^. Subsequently, McEllin and colleagues reported reduced HR-mediated repair and sensitivity to campothecin (CPT) and PARPi in PTEN-deficient cells^19^. However, in the same study they demonstrated that the PTEN defect is associated with radioresistance, which does not fit with the proposed HR deficiency. Moreover, Fraser and colleagues described no effect for PTEN knockdown on HR efficiency or sensitivity to cisplatin or CPT. They did, however, report a significant increase in the sensitivity of PTEN deficient cells to some DNA damaging agents which require HR to repair the induced damage such as PARPi, IR and MMC^16^.

The data presented in the current study provide strong evidence for the involvement of PTEN in DSB repair via HR, based on experiments with four different cell lines. We showed that HR is suppressed in PTEN-depleted cells as evidenced by (i) a suppression of HR efficiency measured by the validated HR repair substrate (Fig. 1B), (ii) an increased sensitivity to MMC as well as the PARPi olaparib (Fig. 1C,D and Supplementary Figure S1C), and (iii) reduced RAD51 foci formation at IR-induced DSBs in PTEN-deficient cells (Fig. 2A). We further reported no association between PTEN status and RAD51 expression either *in vitro* in PTEN-depleted cells (Fig. 3A) or *in vivo* in PTEN-deleted PC patients (Fig. 3B). This excludes the possibility that PTEN regulates HR through controlling the RAD51 expression level. This finding contradicts published data of Shen, Mendes-Pereira or McEllin^18–20^ but is consistent with other data published by R. Bristow’s lab and Gupta and colleagues^16,22^.

Importantly, we reveal here that PTEN depletion and the concomitant sustained activation of AKT (Supplementary Figure S4) cause CHK1 to be sequestered in the cytoplasm (Fig. 4D). Consequently, the CHK1 level available in the nucleus for phosphorylation and activation by ATM/ATR^23^ is reduced, thus impairing G2/M checkpoint control after IR (Fig. 4A–C). These data therefore implicate PTEN in maintaining genomic stability by allowing time for DNA repair by HR before cells enter mitosis. In line with this, we reported for the first time that AKT inhibition recovers the G2/M checkpoint (Fig. 4C–D) and consequently rescues HR efficiency (Fig. 5) in PTEN-depleted cells. In agreement with the involvement of PTEN in cell cycle checkpoint regulation, it has been found that caffeine treatment increased IR-induced chromosomal aberrations and the mitotic index especially in PTEN-deficient cells^22^. Moreover, loss of PTEN was shown to partially impair the CHK1-mediated checkpoint in response to irradiation^22^.

The discrepancies in the published data concerning the role of PTEN in HR can be attributed to the fact that PTEN loss is a late event during carcinogenesis (e.g. in prostate cancer)^28^, suggesting that additional genomic alterations may occur in a PTEN-independent manner which might mask the role of PTEN in HR. That is why, when PTEN was manipulated experimentally, its role in HR could be highlighted and measured. In line with this assumption we observed that, although the PTEN-knockout PC3 cells showed a modest defect in G2/M checkpoint which is reverted upon AKT inactivation (Supplementary Figure S3), they still show no HR deficiency phenotype^5^.

The findings of the current study are potentially of clinical importance for PC patients because of the high frequency of PTEN loss in human PC. Previously, we showed that PTEN deletion correlates with survival of PC patients, being a marker for worse prognosis^25^. Here, we showed that PTEN loss can potentially be used to predict the response of PC patients to radiotherapy (Fig. 6D), though multivariate analysis indicates that it may not be an independent predictive marker (Fig. 6E). A substantial number of PC patients receive IR as a part of their treatment course. Research in recent years has focused on the identification of targets and/or mutations unique to tumor cells, which enable their selective radiosensitization. One important example of such a combined therapeutic approach is the use of PARPi to potentiate the effect of IR. The current study provides some evidence for the potential use of PTEN as a biomarker for predicting the response to PARP inhibitors as radiosensitizing agents in prostate cancer.

## Methods

### Cell culture and treatment

All cell lines used in the current study were obtained from the American Type Culture Collection (ATCC, Manassas, VA, USA). Cell lines were grown in DMEM (Gibco–Invitrogen) supplemented with 10% fetal calf serum, 100 U/ml penicillin and 100 μg/ml streptomycin at 37 °C with 10% CO2. 800 µg/ml G418 or 1 µg/ml puromycin were added to the human cervical carcinoma cell lines HeLa-pEJ (harboring the end joining substrate) and HeLa-pGC (containing the gene conversion substrate), respectively. Irradiation was performed as previously described (200 kV, 15 mA, additional 0.5 mm Cu filter at a dose rate of 0.8 Gy/min)^29^. For inhibition of PTEN, 2 µM of SF1670 (Sigma) or 14 nM of bpv(HOpic) (Sigma) were added to the cells as indicated. 5 µM CAS612847-09-3 (Calbiochem) was used to inhibit AKT activity. CHK1 was inhibited using 50 nM UCN-01 (Sigma). Cells were incubated with 1 µM olaparib (Selleckchem, AZD2281) for 4–6 hours to inhibit PARP activity.

### siRNA transfection

siRNAs employed in this study were a part of a commercially available siGENOME SMART pool (Dharmacon). A final siRNA concentration of 200 nM was transfected using Lipofectamine RNAiMAX (Invitrogen) according to the manufacturer’s protocol. pMKO.1 puro PTEN shRNA was a gift from William Hahn (Addgene plasmid # 10669). Stable cell lines integrating PTEN shRNA or empty vector were selected in puromycin containing media.

### Western blot analysis

Whole cell lysates were subjected to Western blot as previously described^30,31^. Immunoblot analysis was performed with the following antibodies: rabbit anti-RAD51 (Gentex, GTX70230), rabbit anti-PTEN mAb (Cell Signaling, 9559), pAKT (Cell Signaling, 9271) as well as mouse anti-beta-actin (Sigma). Membranes were developed using LiCor Biosciences at room temperature. UNSCAN-IT V6.1 was used to quantify band intensity.

### Plasmid assay

HeLa cells containing single stably integrated copies of homologous recombination repair substrate pGC were transfected with 1 μg of the I-Sce-I expression vector (pCMV3xnls-I-SceI, a kind gift of M. Jasin) to induce DSBs or with pCMV-Neo as a control, as described in manufacturer’s protocol. 24 and 48 h after transfection, the cells were assessed for green fluorescence by flow cytometry (FACS CANTO 2, BD Bioscience). All subsequent repair results were corrected according to the transfection efficiency of each cell line.

### Immunofluorescence

Immunofluorescence analyses were performed as previously described^32^. Briefly, cells grown on coverslips were washed once with cold PBS and fixed with 4% para-formaldehyde/PBS for 10 min. Fixed cells were permeabilized with 0.2% Triton X-100/PBS on ice for 5 min. The cells were incubated overnight with primary antibodies: phospho-S139-H2AX (Millipore, 23464), RAD51 (Calbiochem, PC130), RPA (Santa Cruz Biotechnology, sc-53496), CHK1 (Abcam, ab22610) or anti-CENPF (Lifespan Biosciences, LS-B276 & LS-B3046). After being washed three times with cold PBS, the cells were incubated for 1 h with secondary anti-mouse Alexa Fluor594 (Invitrogen) at a dilution of 1:500 or anti-rabbit Alexa Fluor488 (Invitrogen) at a dilution of 1:600. The nuclei were counterstained with 4′-6-diamidino-2-phenylindole (DAPI, 10 ng/ml). Slides were mounted in Vectashield mounting medium (Vector Laboratories). Immunofluorescence was observed with a Zeiss AxioObserver.Z1 microscope (objectives: ECPlnN 40×/0.75 DICII, resolution 0.44 μm; Pln Apo 63×/1.4 Oil DICII, resolution 0.24 μm; EC PlnN 100×/1.3 Oil DICII, resolution 0.26 μm and filters: Zeiss 43, Zeiss 38, Zeiss 49). Semi-confocal images were obtained using the Zeiss Apotome, Zeiss AxioCamMRm and Zeiss AxioVision Software. Analysis of foci intensities was performed using ImageJ software.

### Colony forming assay

Survival fractions after different treatments were measured using colony forming assay as previously described^5^. 200 cells were seeded in 6-well plates in triplicates, treated as indicated and maintained under optimum culture conditions for up to 3 weeks before staining with 1% crystal violet. In order to examine the effect of PARP inhibition on radiosensitivity, cells were seeded in the presence of PARPi in triplicates and then incubated for 6 h before X-irradiation (RS225 research system, GLUMAY MEDICAL, UK at 200 kV, 15 mA). Colonies containing more than 50 cells were counted as survivors. DMSO was used as a control instead of the inhibitor at the same concentration.

### Cell cycle analysis

Cells were harvested and fixed with 80% cold ethanol (−20 °C). After washing, the DNA was stained with propidium iodide solution containing RNase A. Cell cycle distribution was monitored by flow cytometry (FACS CANTO 2, BD Bioscience) and analyzed using Mod-Fit software (Verity Software House).

### Patients

A preexisting prostate cancer TMA was used for the current study^33^. It consisted of tissue spots from radical prostatectomy specimens from 3,261 patients. All patients were treated by open (ORP) or robotic-assisted (RARP) radical prostatectomy (RP) between 1992 and 2012 at the Department of Urology and the Martini Clinic, University Medical Center Hamburg-Eppendorf, between 1992 and 2004.

20 years follow-up data were available for 2,385 patients undergoing radical prostatectomy. One 0.6 mm tissue core had been punched out from the index tumor of each case, and transferred into a TMA format as described^34^. Prostate-specific antigen (PSA) values were measured following surgery and PSA recurrence was defined as a postoperative PSA of 0.2 ng/mL and increasing after first appearance. All patients with a PSA increase of 0.2 ng/ml or higher after RP were treated by radiation therapy (RT). Patients who received additional androgen deprivation therapy were excluded from analysis. Overall, 778 patients were included in the study for whom information of PTEN status was available. PSA progression after RT was defined as a PSA level of ≥0.2 ng/mL.

### Fluorescence *in Situ* Hybridization (FISH)

FISH was used to detect genomic PTEN deletions in 778 patients (expanded from)^25^ as previously described^35^. In brief, a dualcolor FISH probe was constructed from two Spectrum Orange-labeled BAC clones (RP11-380G05, RP11-813O03; Source Bioscience, Nottingham, UK) and a commercial Spectrum Green-labeled centromere 10 (CEP10) reference probe (Abbott Molecular, Wiesbaden, Germany).

The stained slides were visually inspected under an epifluorescence microscope as previously described^35^. Briefly, at least 30 different tumor cell nuclei were analyzed for the FISH signal numbers per tissue spot. PTEN deletion was defined as follows: homozygous PTEN deletion was considered if PTEN deletion probe signals were completely lacking in >60% of tumor nuclei, while PTEN FISH signals were present in adjacent normal cells. Heterozygous deletion of PTEN was defined as presence of fewer PTEN signals than centromere 10 probe signals in >60% of tumor nuclei.

### Immunohistochemistry

Freshly cut TMA sections were deparaffinized and exposed to heat-induced antigen retrieval for 5 minutes in an autoclave at 121 °C in pH 7.8 Tris-EDTA-Citrate buffer. RAD51 (US Biological, clone 3F326) was applied at 37 °C for 60 minutes. Bound antibody was then visualized using Diaminobenzidine as a chromogen. A final score was built from these two parameters according to the following criteria as previously described^36^: Tumors with complete absence of staining were scored as “negative”. Tumors with a score “weak” had a staining intensity of 1+ in ≤70% of tumor cells or 2+ in ≤30% of tumor cells. A score “moderate” was given to cancers with a staining intensity of 1+ in >70% of tumor cells, or 2+ in >30% and ≤70% of tumor cells, or 3+ in ≤30% of tumor cells. The score was “strong” if staining intensity was 2+ in >70% of tumor cells or 3+ in >30% of tumor cells.

### Graphs and statistics

Unless stated otherwise, experiments were independently repeated at least three times. Data points represent the mean ± SEM of all individual experiments. Statistical analysis, data fitting and graphics were performed with the GraphPad Prism 6.0 program (GraphPad Software) and R^37^.

### Ethical approval

This study was in accordance with the World Medical Association Declaration of Helsinki and the guidelines for experimentation with humans by the Chambers of Physicians of the State of Hamburg (“Hamburger Ärztekammer”). All patients have given their authorization for research purposes by signed informed consent. All experiments were approved (Approval No. PV-3652) by the Ethics Committee of the Chambers of Physicians of the State of Hamburg (“Hamburger Ärztekammer”).

### Data availability

All datasets are available from the authors upon reasonable request.

## Electronic supplementary material

Supplementary Figures
