# Supplementary material for: Loss of PTEN-assisted G2/M checkpoint impedes homologous recombination repair and enhances radio-curability and PARP inhibitor treatment response in prostate cancer
Source: Sci Rep. 2018 Mar 2;8:3947. doi: 10.1038/s41598-018-22289-7 (PMC5834544; doi:10.1038/s41598-018-22289-7)
Supplement: Supplementary file 1 — Supplementary Figures [file 41598_2018_22289_MOESM1_ESM.pdf]

Loss of PTEN-assisted G2/M checkpoint impedes homologous recombination repair and enhances radio-curability and PARP inhibitor treatment response in prostate cancer

W. Y. Mansour, P. Tennstedt, J. Volquardsen, C. Oing, M. Kluth, C. Hube-Magg, K. Borgmann, R. Simon, C. Petersen, E. Dikomey, K. Rothkamm

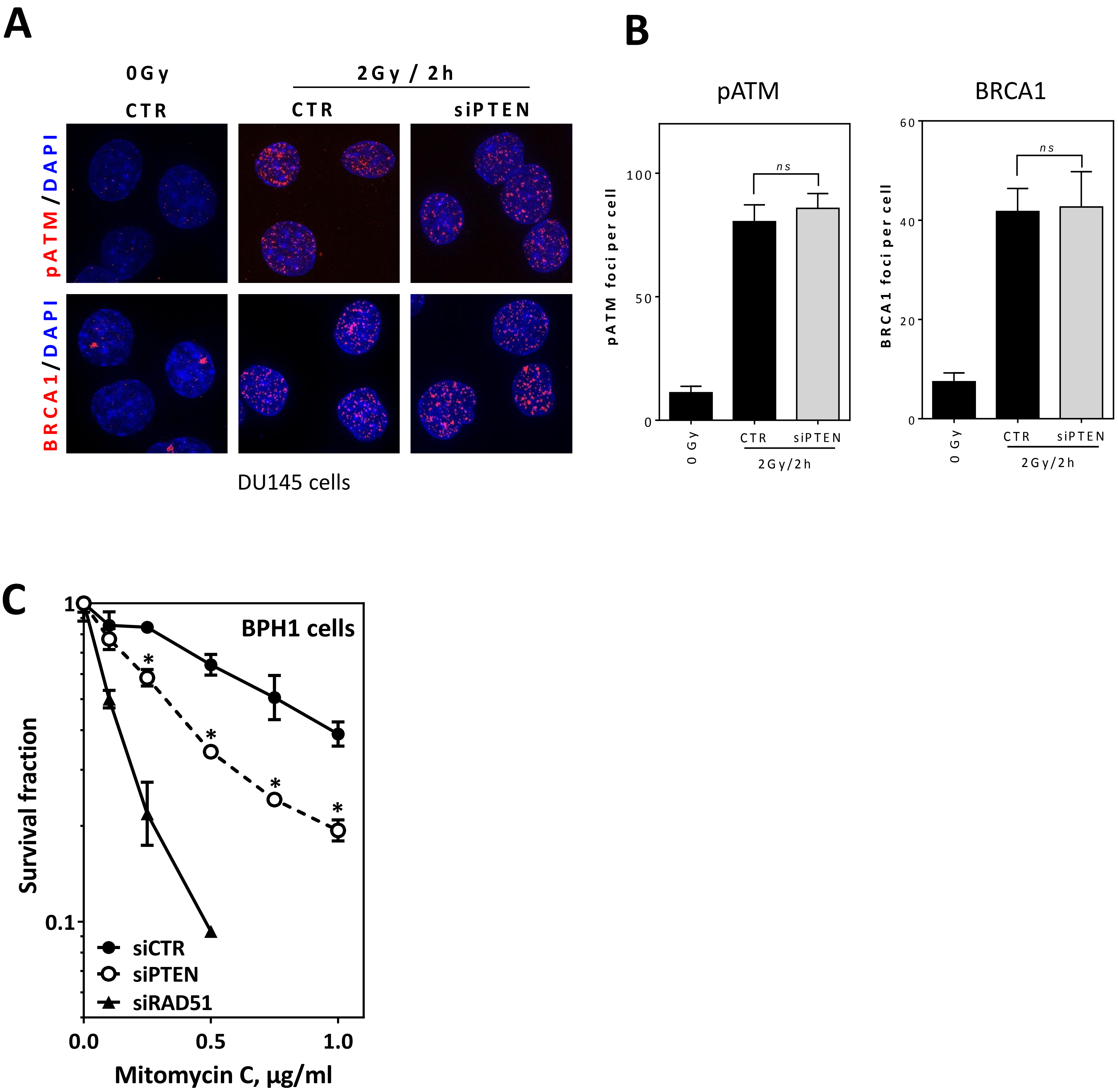

Supplementary Figure S1. PTEN-depletion impairs the repair of DSB via HR. (A) Representative micrographs for pATM and BRCA1 foci at 2h after 2Gy in PTEN depleted cells compared to their wildtype counterparts. (B) Quantitation of the experiment conducted in A. (C) Survival fractions after treatment with the indicated concentrations of mitomycin C were measured in mock- (siCTR) or PTEN- depleted (siPTEN) DU145 using colony forming assay. As a positive control in all experiments, RAD51 was depleted using siRNA (siRAD51). Shown are mean ±SEM of three independent experiments. ns: not statistically significant

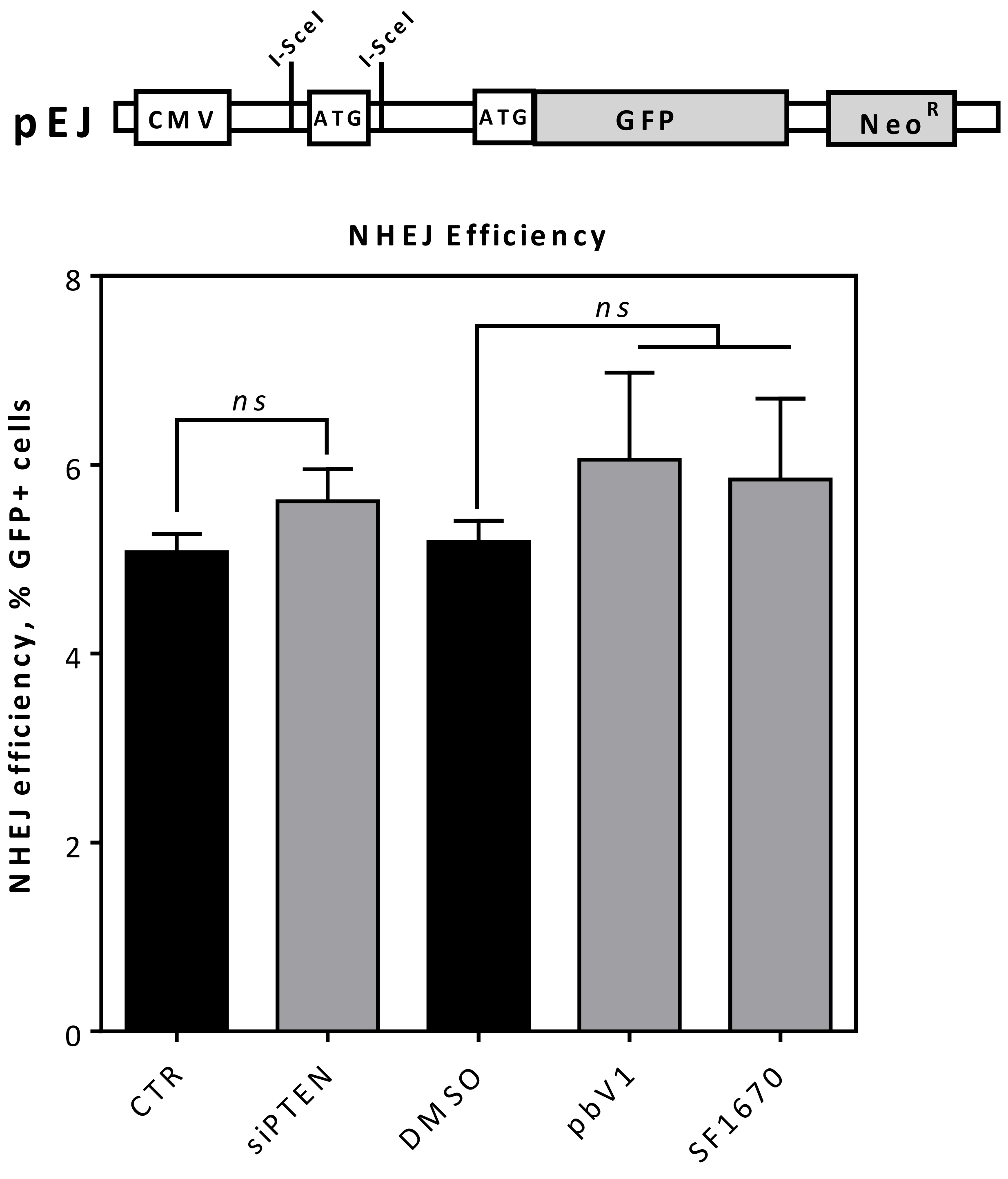

Supplementary Figure S2. NHEJ is not affected by PTEN depletion. HeLa cells harboring single copies of NHEJ substrate pEJ were transfected with scRNA (siCTR) or siRNA against PTEN (siPTEN) for 24h before transfection with I-SceI-expression vector to induce DSB. The percent of GFP+ cells was then measured after 24h using flow cytometry as an indication of NHEJ efficiency. For inhibition of PTEN, cells were incubated with 14nM bpVHOpic or 2µM SF1670 for 6h before induction of DSB. Shown are means ±SEM of three independent experiments. ns: not statistically significant

A

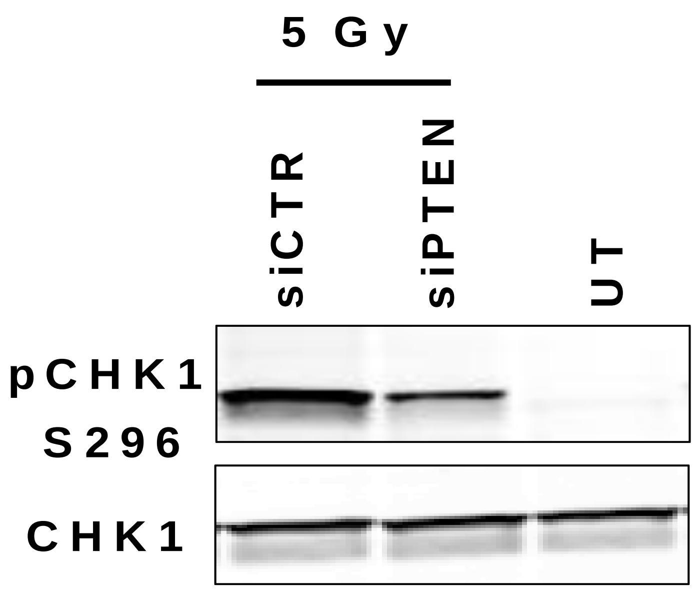

B

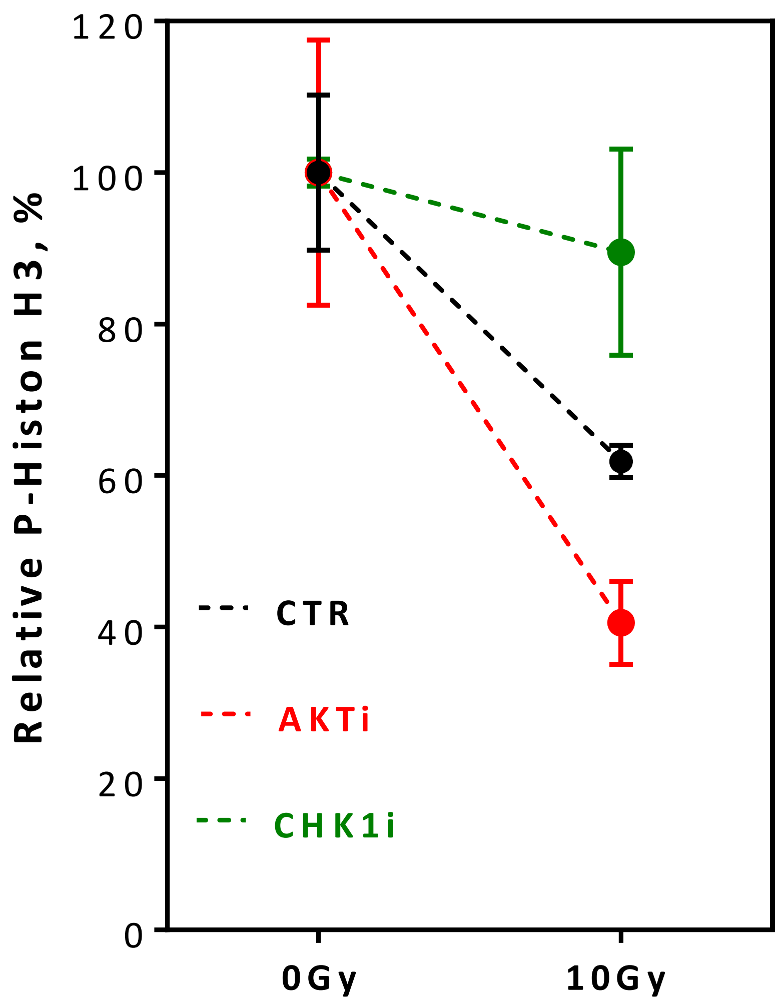

Supplementary Figure S3. (A) DU145 cells were transfected with either scrambled (CTR) or PTEN (siPTEN) siRNA. After 48h, cells were either mock irradiated (UT) or irradiated with 10Gy and the CHK1 auto-phosphorylation site (S296) was immunoblotted. CHK1 was used as loading control. (B) PC3 cells were treated with or without 10Gy and after 2h, the percentage of phosphor-histone H3 (S10), as a marker for mitotic cells, was measured after the indicated treatments. Cells were treated with colcemid to accumulate mitotic cells. Shown are the means ± SEM from three experiments.

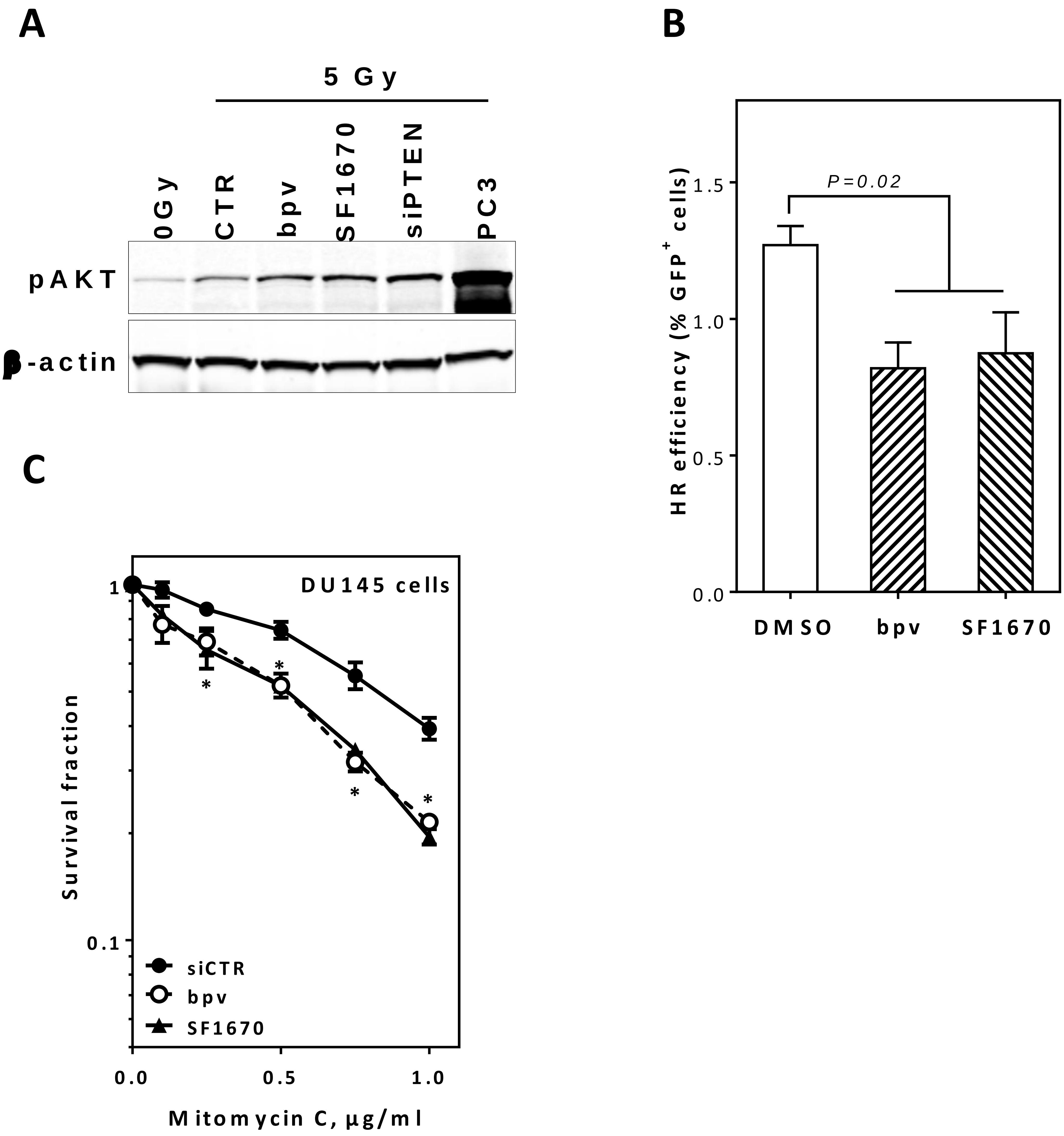

Supplementary Figure S4. (A) Western blot showing pAKT at S473 (autophosphorylation site) after the indicated treatments. For inhibition of PTEN, cells were incubated with either 14nM bpVHOpic or 2µM SF1670 for 4h. (B) Homologous recombination was measured in HeLa cells harboring single copies of pGC substrate as described in Fig.1 after the indicated treatments. (C) Mitomycin-C sensitivity in DU145 cells treated with the indicated PTEN inhibitor. Shown are the means ± SEM from three experiments. Asterisk (*) represents significant difference (P<0.05).

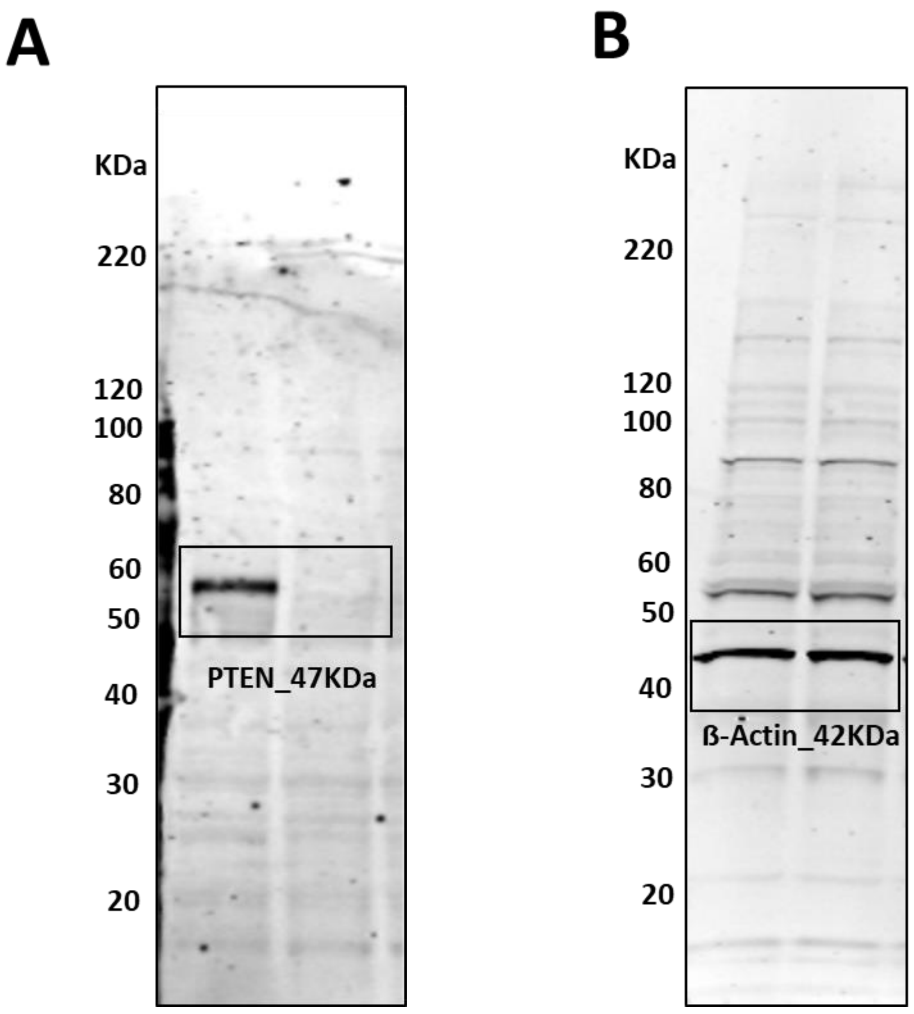

Supplementary Figure S5. The full length blots of Figure 1B. Crop lines are indicated in the full length western blots for (A) PTEN or (B) β-actin. After reaction with the anti-PTEN antibody, the same blot was stripped, re-blocked with skim milk and then reacted with the anti-β-actin antibody. KDa; Kilodalton.

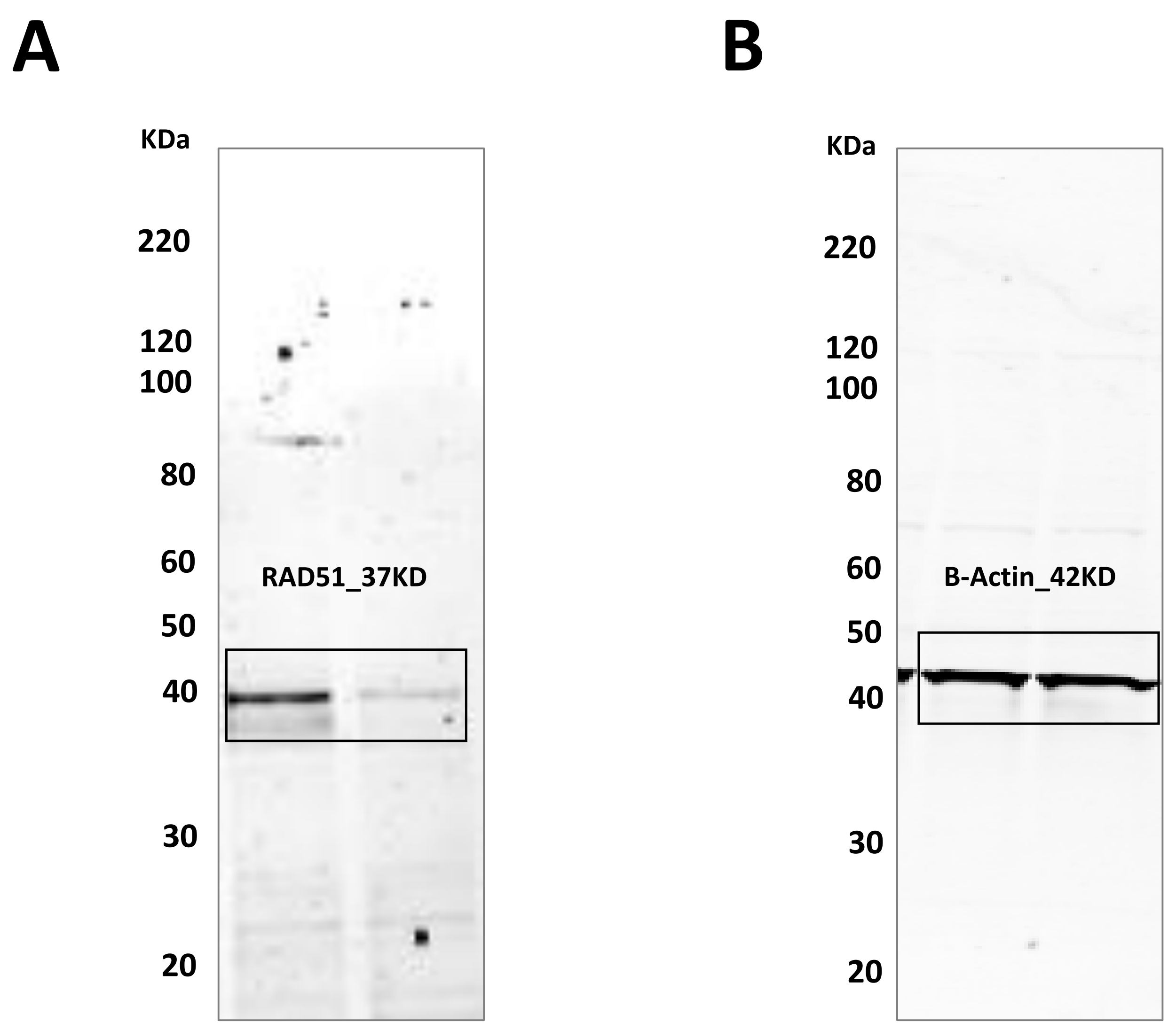

Supplementary Figure S6. The full length blots of Figure 1B. Crop lines are indicated in the full length western blots for (A) RAD51 or (B) β-actin. After reaction with the anti-RAD51 antibody, the same blot was stripped, re-blocked with skim milk and then reacted with the anti-β-actin antibody. KDa; Kilodalton.

A

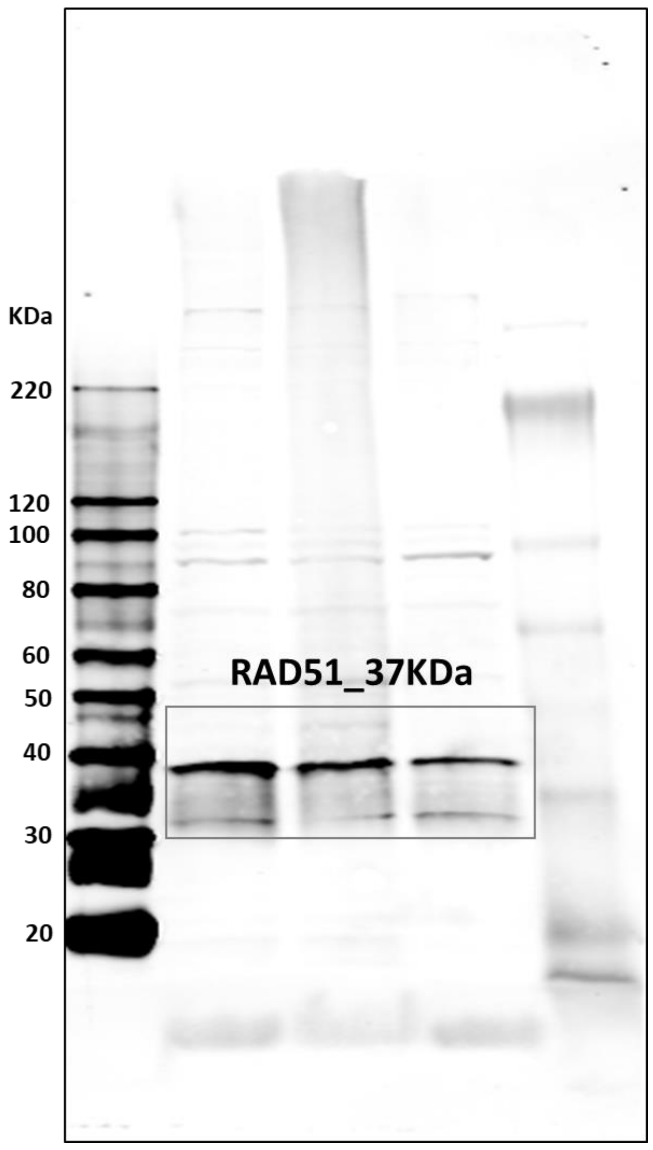

B

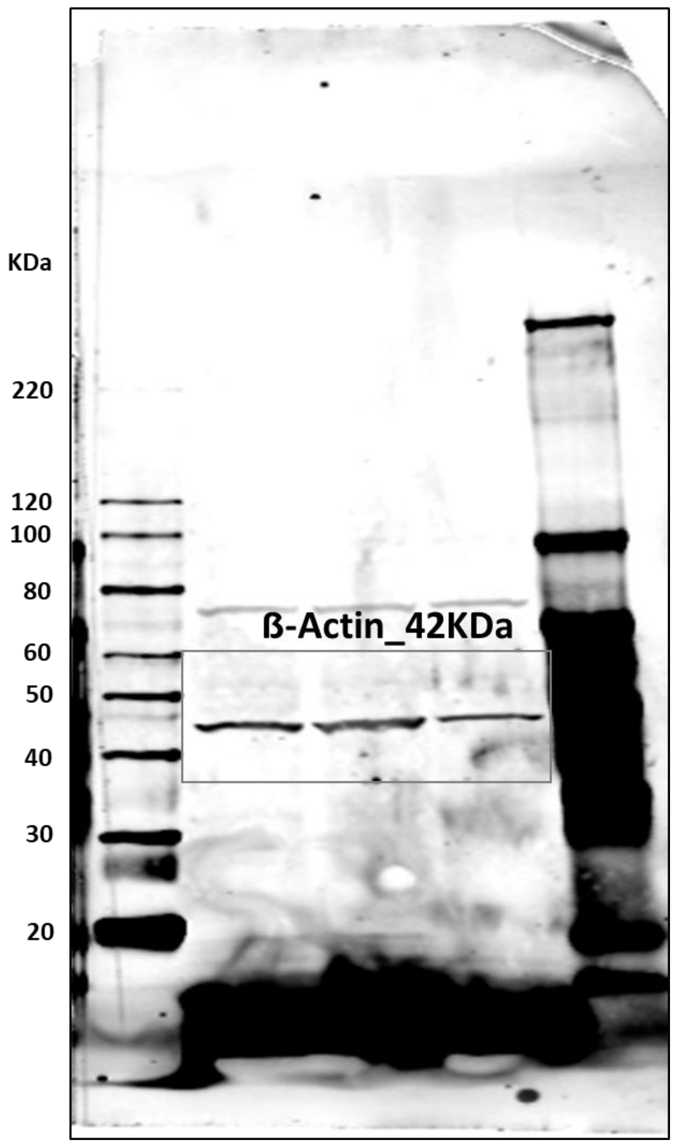

Supplementary Figure S7. The full length blots of Figure 3A. Crop lines are indicated in the full length western blots for (A) RAD51 and (B) β-actin. After reaction with the anti-RAD51 antibody, the same blot was stripped, re-blocked with skim milk and then reacted with the anti-β-actin antibody. KDa; Kilodalton.

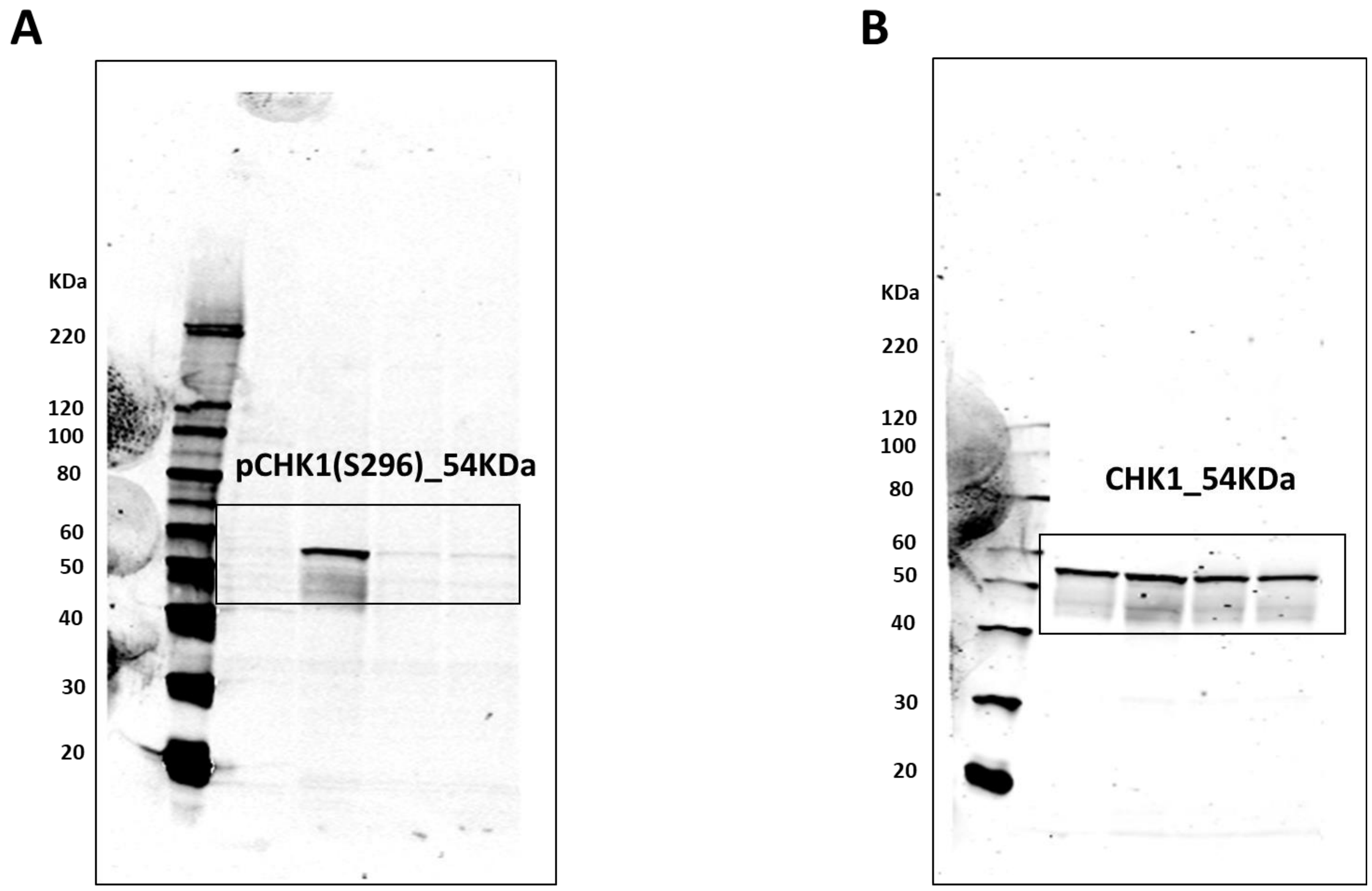

Supplementary Figure S8. The full length blots of Figure 4A. Crop lines are indicated in the full length western blots for (A) pCHK1-S296 and (B) CHK1 in DU145 cells. After reaction with the anti-pCHK1 antibody, the same blot was stripped, re-blocked with skim milk and then reacted with the CHK1 antibody. KDa; Kilodalton.

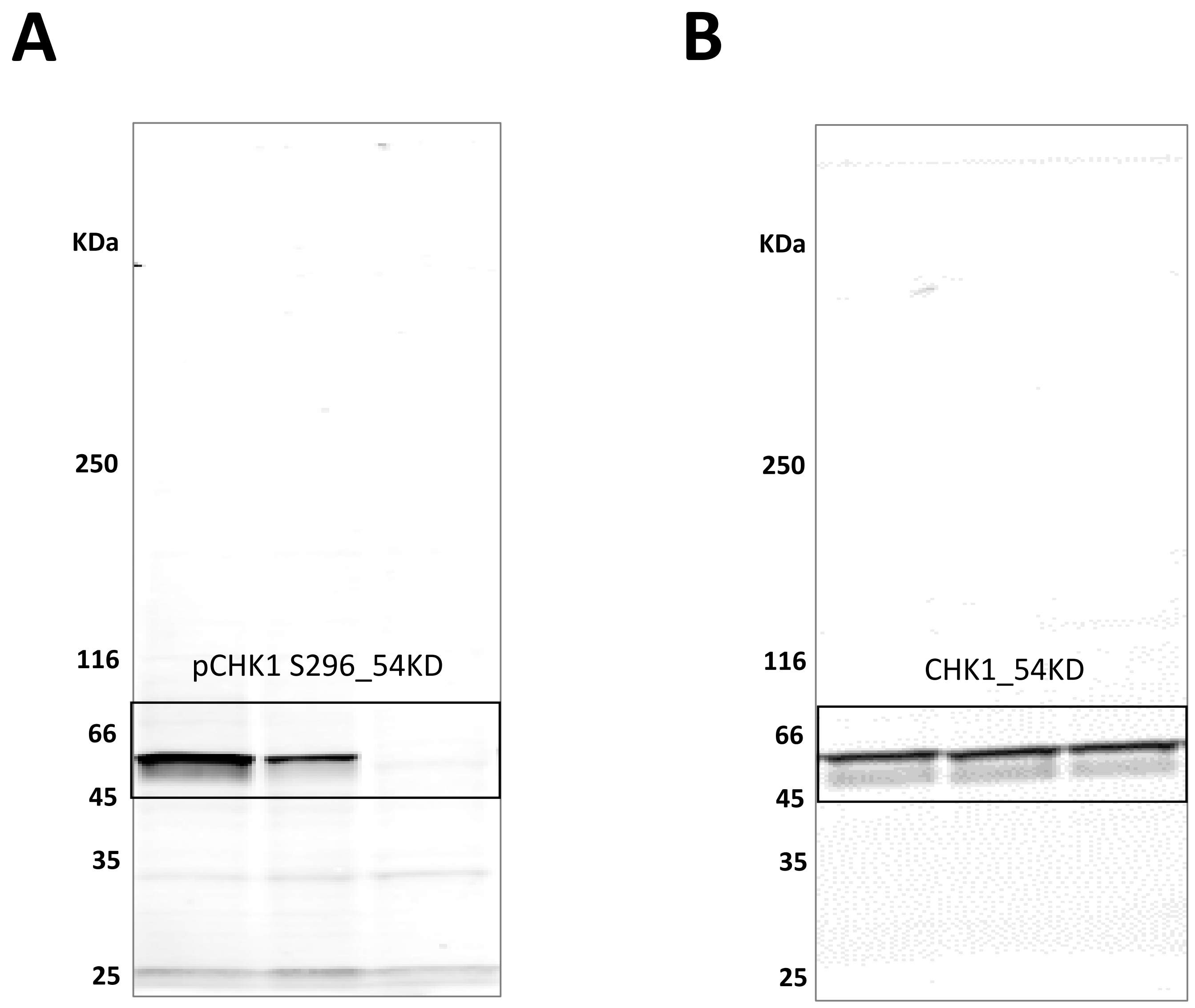

Supplementary Figure S9. The full length blots of Supplementary Figure S3A. Crop lines are indicated in the full length western blots for (A) pCHK1-S296 and (B) CHK1 in DU145 cells. After reaction with the anti-pCHK1 antibody, the same blot was stripped, re-blocked with skim milk and then reacted with the CHK1 antibody. KDa; Kilodalton.

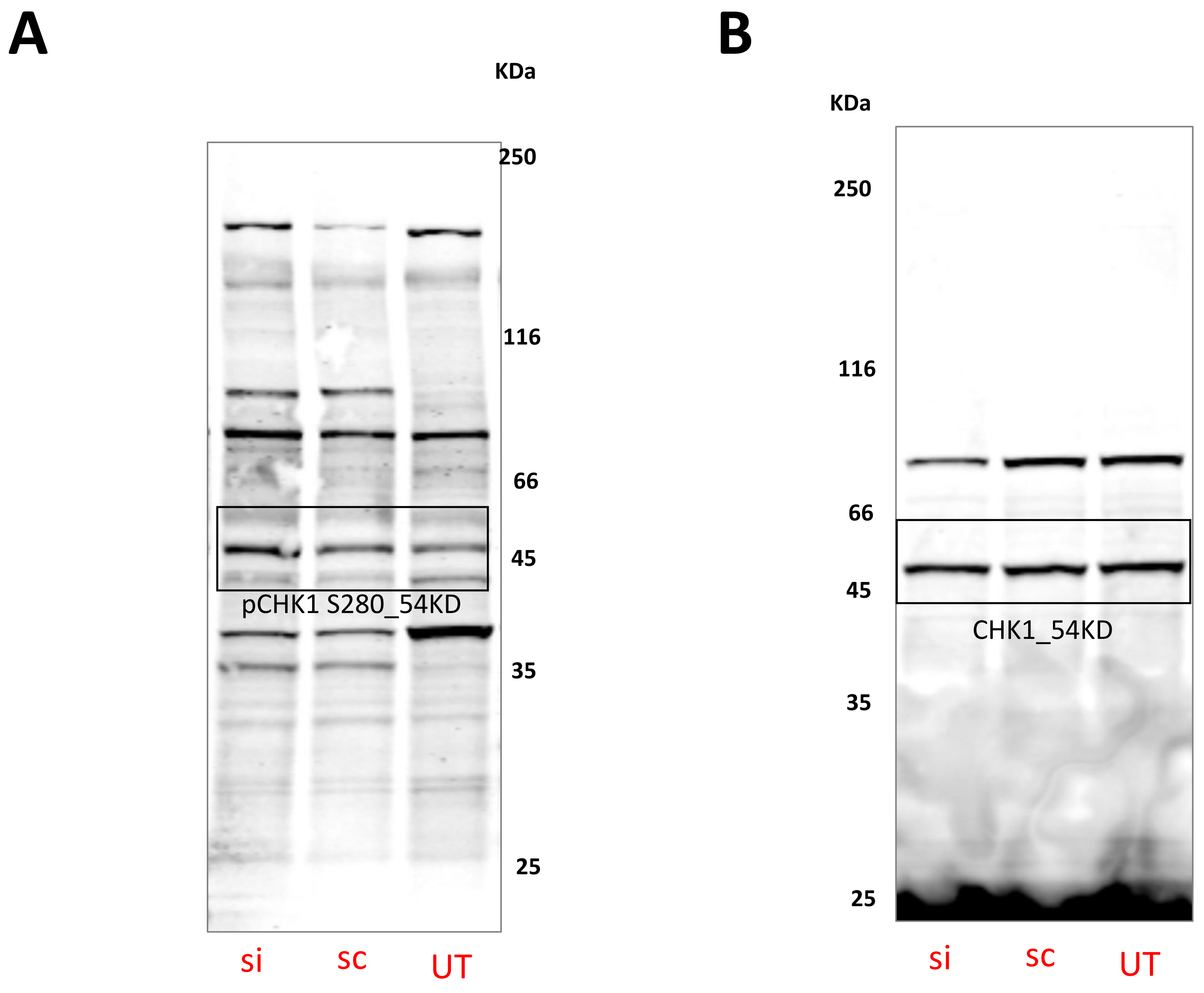

Supplementary Figure S10. The full length blots of Figure 1F. Crop lines are indicated in the full length western blots for (A) pCHK1-S280 and (B) CHK1 in DU145 cells. After reaction with the anti-pCHK1 antibody, the same blot was stripped, re-blocked with skim milk and then reacted with the CHK1 antibody. KDa; Kilodalton. si: siPTEN; sc: siControl; UT: untreated

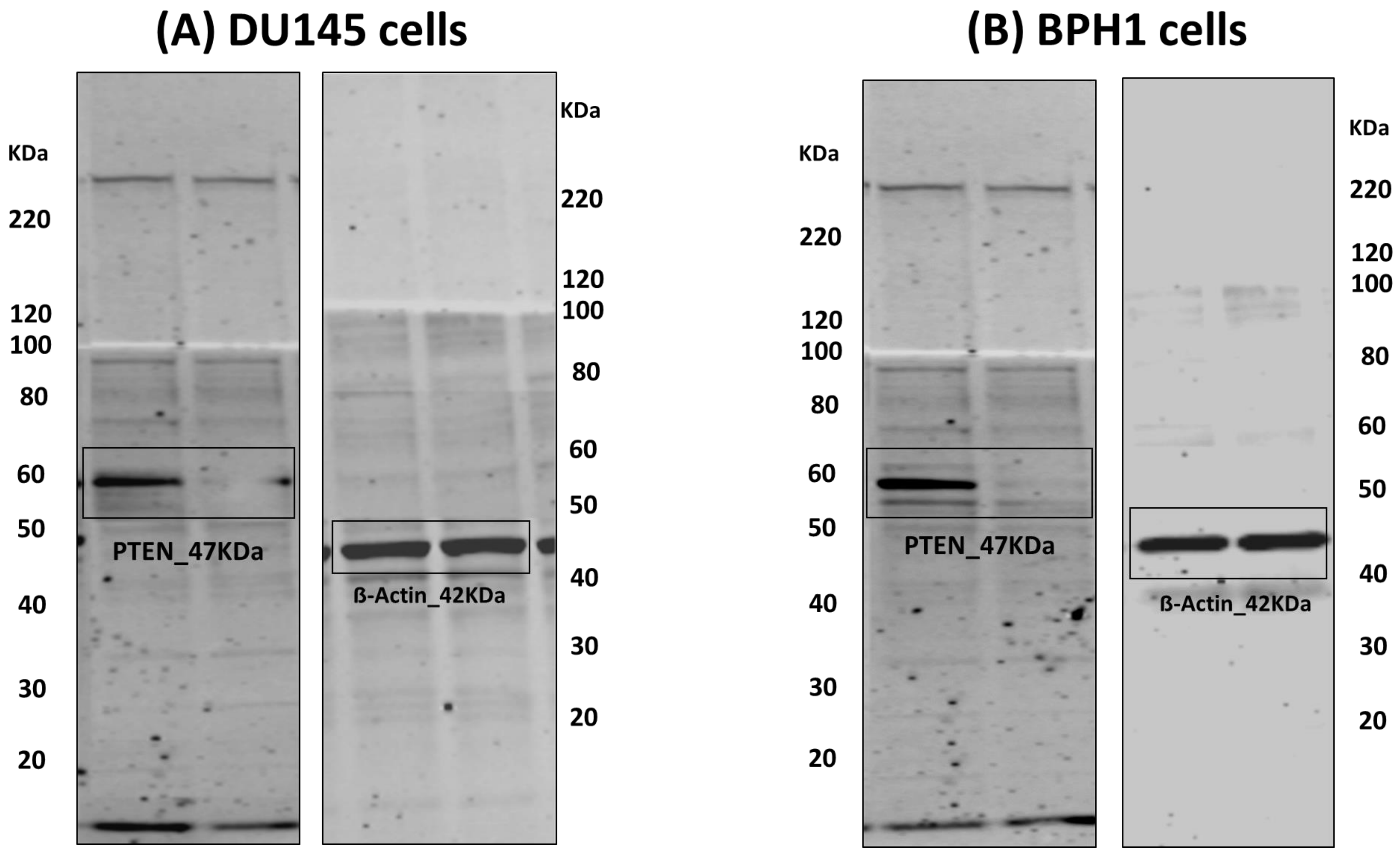

Supplementary Figure S11. The full length blots of Figures 6A&B. Crop lines are indicated in the full length western blots for PTEN and β-actin in (A) DU145 or (B) BPH1 cells. After reaction with the anti - PTEN antibody, the same blot was stripped, re-blocked with skim milk and then reacted with the anti-β-actin antibody. KDa; Kilodalton.

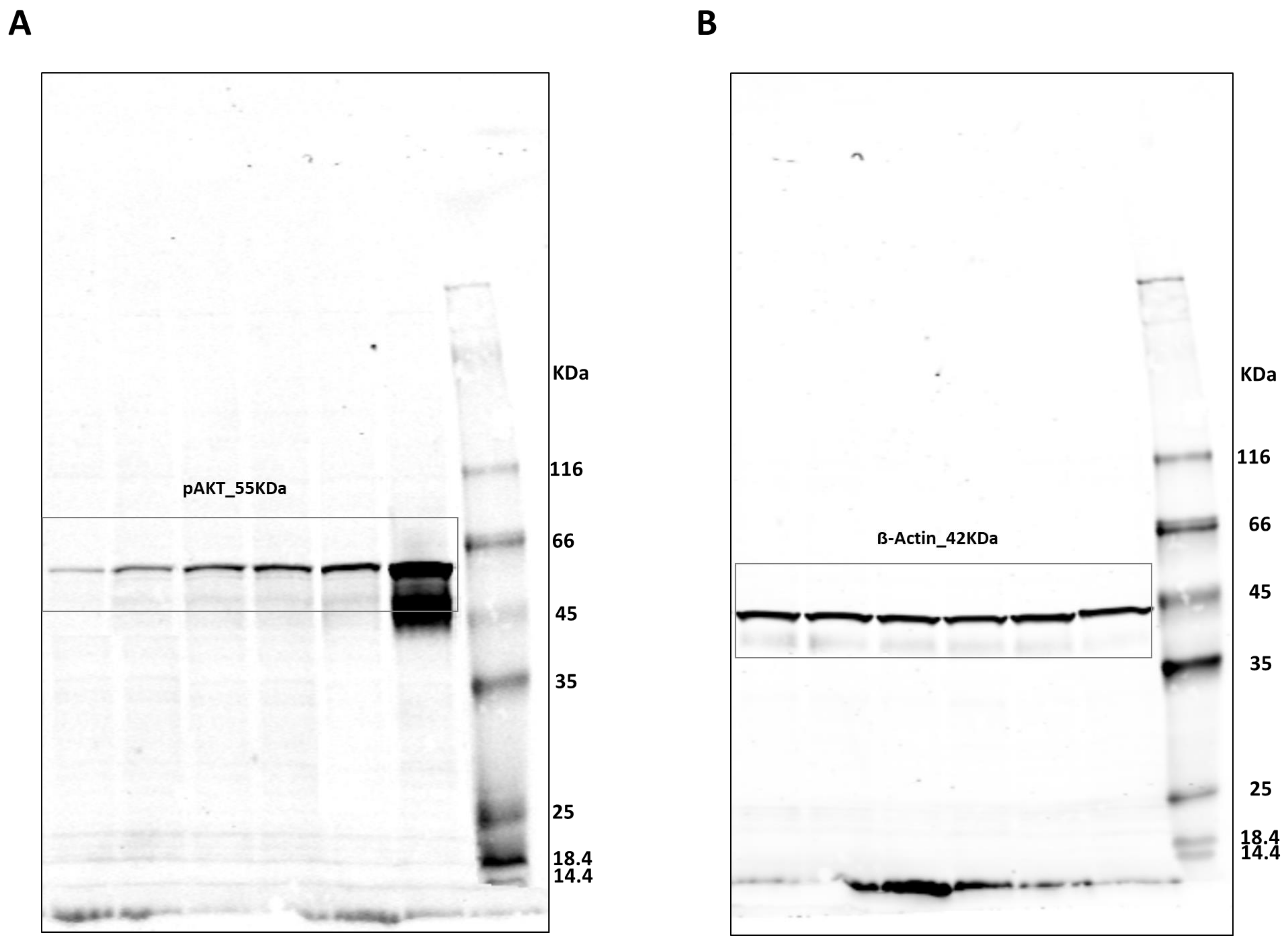

Supplementary Figure S12. The full length blots of Supp. Figure S4A. Crop lines are indicated in the full length western blots for (A) pAKT or (B) β-actin. After reaction with the anti-pAKT antibody, the same blot was stripped, re-blocked with skim milk and then reacted with the anti-β-actin antibody. KDa; Kilodalton
